# Tacrolimus-induced epilepsy with primary membranous nephropathy: A case report

**DOI:** 10.1097/MD.0000000000024989

**Published:** 2021-03-05

**Authors:** Yan Yang, Lei Zhang, Ying Mo, Rong Ren, Fengmei Wang

**Affiliations:** a Institute of Nephrology, Zhong Da Hospital, Southeast University School of Medicine, Nanjing; b Department of Nephrology, The fifth Affiliated Hospital of Xinjiang Medical University, Urumqi, Xinjiang, China.

**Keywords:** case report, epilepsy, primary membranous nephropathy, tacrolimus

## Abstract

**Rationale:**

Tacrolimus-associated neurologic disorders can be found in some cases, mainly in organ transplantation patients. However, epilepsy induced by tacrolimus in primary membranous nephropathy (PMN) patient is scare.

**Patient concerns:**

A 63-year-old man experienced 1-year history of foamy urine, and edema of lower extremity.

**Diagnosis:**

The patient had proteinuria, hypoalbuminemia, which indicated nephrotic syndrome. Further, we performed renal biopsy for this patient. Combined with the renal biopsy result, the diagnosis of primary membranous nephropathy was established.

**Intervention:**

At first, irbesartan was administrated for 6 months. However, the proteinuria had no obvious improvement. Tacrolimus was administrated afterwards.

**Outcomes:**

Twenty-two days after tacrolimus treatment, epilepsy occurred. Sodium valproate and carbamazepine were successively given to control epilepsy. However, the epileptic symptoms were not effectively controlled. During the treatment, the concentration of tacrolimus fluctuated greatly. At last, levetiracetam was given to maintain the curative effect. Fortunately, the patient did not suffer from epilepsy again. The concentration of temporary tacrolimus was stable, whereas proteinuria gradually decreased.

**Lessons:**

Tacrolimus-induced epilepsy should be considered in patients exhibiting acute neurological symptoms. Early diagnosis and effective treatment play a vital role for favorable prognosis.

## 1 Introduction

Primary membranous nephropathy (PMN) is an immune-mediated cause of nephrotic syndrome. In 2009, Beck et al^[1]^ found M-type phospholipase A2 receptor (PLA2R) was colocalization with IgG in glomeruli of PMN. PLA2R-antibodies (Abs) can be detected in serum of 70% of PMN patients. In 2014, Tomas et al^[2]^ discovered 8% to 14% PMN patients with thrombospondin Type I domain-containing 7A (THSD7A) antibody positive, whereas with negative PLA2R-Ab in serum. Because spontaneous remission is relatively common in PMN and immunosuppressive treatment has adverse effects, it is important to assess the risk of progressive loss of kidney function prior to determine whether and when to implement immunosuppressive treatment. When patients present with deteriorating renal function, rituximab, cyclophosphamide or calcineurin inhibitors such as cyclosporine and tacrolimus may be considered for immediate immunosuppressive therapy.^[3]^

KDIGO guideline in 2020 and other literatures have indicated that tacrolimus is safe and effective for patients with PMN. However, in clinical practice, common adverse events following tacrolimus such as gastrointestinal disorders, endocrine abnormalities, infection, and hematological abnormalities can occur. Occasionally, tacrolimus-associated neurologic disorders, including common confusion, somnolence, cortical blindness, epilepsy, uncommon coma, could be found in some organ transplantation cases.^[4–6]^ Herein, we report a rare case of epilepsy induced by concentration fluctuations of tacrolimus in a PMN patient, who was recovered after therapy with levetiracetam. To the best of our knowledge, this is the first case report that tacrolimus-induced epilepsy occurred in a patient with PMN.

## 2 Case report

A 63-year-old man presented to our hospital with 1-year history of foamy urine, and edema of lower extremity in May 2019. He had a history of hypertension for 2 years, chronic atrial fibrillation for 1 year, and cerebral infarction for 3 months. On examination, his blood pressure was 120/80 mm Hg, accompanying with atrial fibrillation rhythm, limb disorders after cerebral infarction, left side deviation, and edema of both lower limbs.

**Figure 1:**
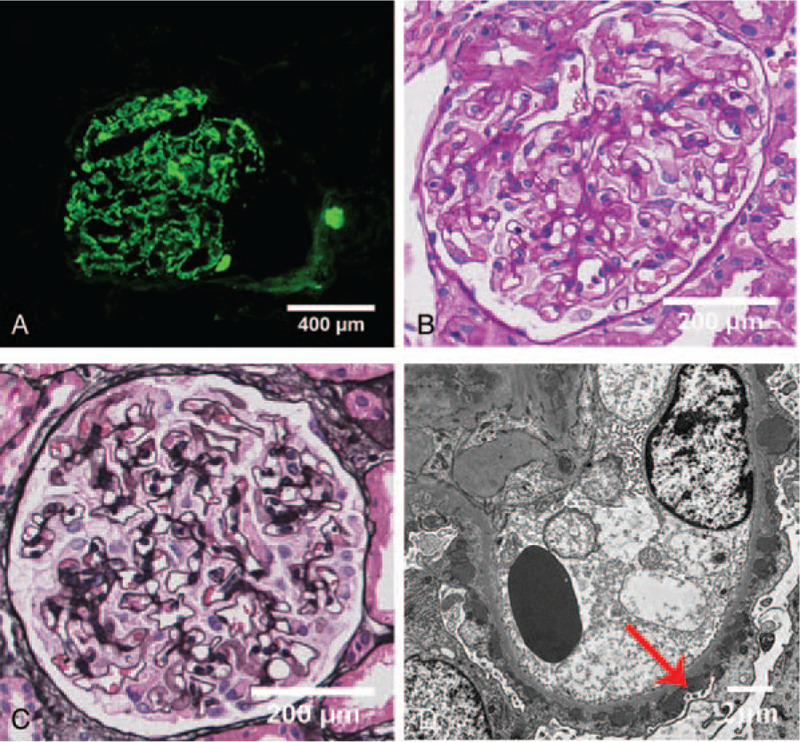
The pathological results of kidney biopsy. (A) Immunofluorescence showed immunoglobin G (IgG) deposited along the glomerular capillary. (B) Periodic Acid-Schiff (PAS) and (C) Periodic Acid-Silver Metheramine (PASM) staining demonstrated discrete subepithelial “spike” formation along all of the glomerular capillaries in this patient. (D) The electron microscopy displayed abundant subepithelial deposits with intervening glomerular basement membrane (GBM) “spikes” (red arrow).

Urinalysis test showed proteinuria levels of 1.56 to 2.7 g/24 h. Biochemistry analysis revealed the level of serum albumin of 29.1 g/L, and serum creatinine level of 89 μmol/L. The abdominal ultrasound, anti-neutrophil cytoplasmic antibodies, anti-GBM antibody, antinuclear antibody, viral hepatitis, and tumor markers were all normal. Interestingly, anti-PLA2R antibody was at high level (110.41 RU/mL). We performed renal biopsy for further diagnosis. Prominent granular deposition of IgG was found along the glomerular capillary by immunofluorescence patterns. The light microscopy demonstrated discrete subepithelial “spike” formation along all of the glomerular capillaries in this patient. The electron microscopy displayed abundant subepithelial deposits with intervening GBM “spikes” (Fig. 1). Eventually, PMN was diagnosed.

**Figure 2:**
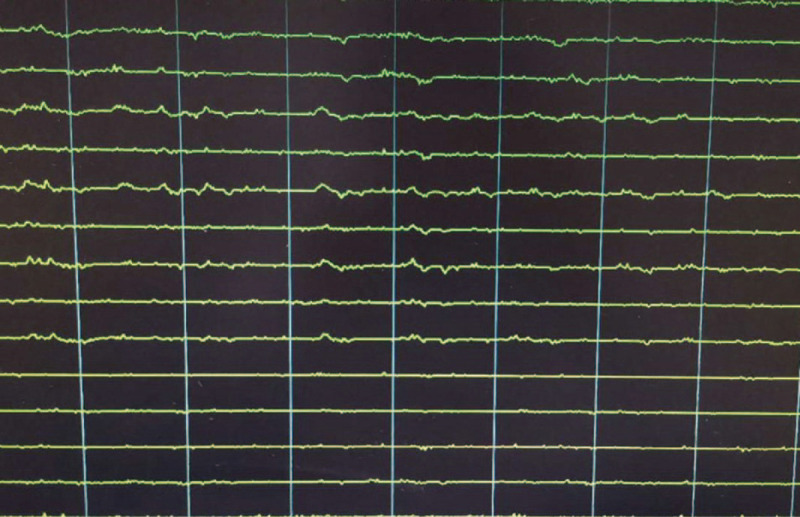
Electroencephalogram revealed diffuse β fast wave activity, with some Q wave activity and some eye movement disturbance.

At first, irbesartan was administrated for 6 months. However, proteinuria and serum albumin had no obvious improvement. On December 24, 2019, tacrolimus (1 mg bid) was administrated. Twenty-two days after tacrolimus treatment, he was admitted to the emergency room. His symptoms were a sudden numbness in his left upper limb, disturbance of consciousness, convulsions in his limbs, and rolling up of his eyes, which lasted for about 10 minutes and then recovered. The magnetic resonance imaging (MRI) disclosed multiple lacunar cerebral infarction with the right temporal and occipital lobe softened lesion formation, leukoaraiosis, and brain atrophy. Electroencephalogram showed diffuse β fast wave activity, with some Q wave activity and some eye movement disturbance (Fig. 2). He was diagnosed as symptomatic epilepsy, then sodium valproate was administrated (0.25 g bid), combined with tacrolimus. In the next 2 days, the patient had complete reversal of neurological symptoms. However, on April 3, 2020, the patient developed epilepsy again. Although the concentration of sodium valproate was normal, which was 128.1 μg/mL (50–100). Carbamazepine (0.2 g bid) was chosen instead of sodium valproate. Unfortunately, on April 21, 2020, epilepsy occurred again. We tested the concentration of carbamazepine, which was also in a normal range (6.2 μg/mL).

Why did this patient suffer from epilepsy again and again under the antiepileptic drug treatment? According to the epilepsy manifestations of patient, neurologist concluded that location diagnosis of epilepsy originated from brainstem reticular structure, thalamus or cerebral cortex, and qualitative diagnosis attributed to vascular injury. Considering the common inducers of epilepsy without new cerebral infarction, abnormal electrolyte, the administration of tacrolimus was suspected. According to previous studies,^[7,8]^ 2 factors might contribute to tacrolimus induced epilepsy higher plasma levels of tacrolimus; the fluctuations of tacrolimus concentration. Firstly, we tested CYP3A5 gene for tacrolimus. Tacrolimus is metabolized by CYP3A5, and its gene polymorphism is an important factor affecting the plasma concentration. The result showed the gene type of CYP3A5 for this patient was AG, which indicated that tacrolimus was intermediate metabolic type. Consequently, we found that tacrolimus concentrations fluctuated greatly from January 7 to April 24. However, it was unclear what caused the fluctuation. We further analyzed the drug interactions to identify possible reasons.

**Figure 3:**
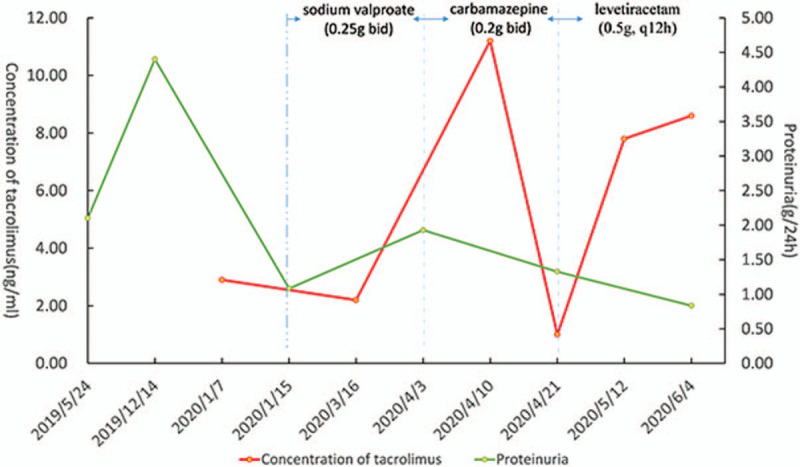
The concentration of tacrolimus and the change of 24 hours urine protein levels for this patient.

Carbamazepine is a CYP3A4 liver enzyme inducer, which can reduce the concentration of tacrolimus (the concentrations of tacrolimus were showed in Fig. 3). Levetiracetam (0.5 g, q12 h) was administered in April 2020, instead of carbamazepine. The drug has a weak interference on cytochrome P450 enzyme, and hardly affects the plasma concentration of tacrolimus. Up to June 2020, the plasma concentration of tacrolimus was 7.8 to 8.6 ng/mL, and fortunately, the patient had not suffered from epilepsy again. The proteinuria gradually decreased (Fig. 3).

## 3 Discussion

Herein, we reported a 63-year-old man who received tacrolimus with PMN. Tacrolimus-induced epilepsy was diagnosed by electroencephalography and clinical symptoms. After the patient was treated with levetiracetam, the plasma concentration of tacrolimus was maintained normally, epilepsy never occurred to this patient.

Immunosuppressants such as cyclosporine A, cyclophosphamide, and tacrolimus have been widely used in PMN.^[3]^ Tacrolimus gradually becomes the first-line therapy among them. However, in recent years, some cases have reported tacrolimus could induce seizures after organ transplant.^[4]^ The prevalence of neurotoxicity induced by calcineurin inhibitor ranges from 10% to 33% among organ transplant recipients.^[5,6]^ Among these neurologic complications, seizures have been reported abort 5% to 10% in transplant patient.^[9]^ In 2019, Li et al^[10]^ reported 2 cases occurred acute symptomatic seizure triggered by tacrolimus after liver transplantation. However, studies about tacrolimus induced epilepsy with nephropathy are scare. In 2004, Loeffler et al^[11]^ reported 16 children received tacrolimus with resistant nephrotic syndrome. They only found 1 patient had a 2-minute generalized tonic-clonic seizure accident after taking tacrolimus for 1 month, who did not need anticonvulsant therapy. In this case, we reported a patient occurred tacrolimus induced epilepsy with PMN with long-term anticonvulsant. This might be due to the patient's older age and cerebrovascular disease.

The exact mechanism on neurotoxicity of calcineurin inhibitors (CNIs) remained unclear. One possible hypothesis is that CNIs may be mediated by upregulating endothelin receptors, damaging blood-brain barrier, and interacting with neuromodulatory systems. An alternative hypothesis is that long term application of CNIs might impair cerebral mitochondrial energy metabolism, leading to neurodegeneration and cognitive impairment. What's more, Zhang et al^[12]^ has found that the complex of CNIs and immunophilins might be related with neurotoxicity. In addition, vasculopathy may also be included in CNI-induced neurotoxicity.^[12]^

One study in rats found that the threshold tacrolimus concentration in the brain triggering neurotoxic events was approximately 700 ng/g, whereas for the whole blood as 20 ng/mL in rats.^[7]^ What is more, Lyson et al^[13]^ demonstrated that tacrolimus-binding protein, calmodulin, and cyclophilic protein were distributed in most brain tissue, and they further confirmed that sympathetic activation which induced by FK-506 associated with calcineurin-mediated inhibition of T-cell signaling in brain. All the evidence suggests that the concentration of tacrolimus in the brain may determine the occurrence of encephalopathy. Further studies have found that encephalopathy symptoms in patients are related with high blood levels of tacrolimus,^[7–8]^ but can also be happened in those with concentration in therapeutic range.^[14]^ The patient in our case had the history of cerebral infarction, hypertension, and volatility of tacrolimus concentration, which maybe cause him to be more susceptible to encephalopathy.

Apart from the case like ours, many studies have found that immunosuppressants can induce reversible posterior leucoencephalopathy syndrome (RPES), which was first reported in 1996.^[15]^ The major clinical manifestations of RPES are headaches, an altered mental status, and seizures with typical imaging changes.^[16]^ One case reported a female patient who received tacrolimus as an immunosuppressive regimen after kidney transplantation. Five weeks after transplantation, she was admitted to the emergency due to RPES, manifested by sudden onset of confusion, disorientation, visual disturbances, and major headache.^[17]^ Another case-control study, including 51 patients receiving tacrolimus, cyclosporine or prednisolone owing to nephrotic syndrome, of these 21 with RPES and 30 without, found that hypertension, proteinuria, hypercholesterolemia, and lower serum albumin levels were more common in RPES patients.^[18]^ Our patient also had these risk factors, but not clear whether is caused by RPES. RPES has classic imaging findings of presence of edema of the gray and white matter in posterior brain, and it can be complete recovery. However, after 4 months follow-up, compared with his cerebral MRI in January 2020, the MRI did not recover.

In our case, the epilepsy was discontinued with levetiracetam instead of other antiepileptic drugs, such as sodium valproate and carbamazepine. Pharmacologically, the effect of sodium valproate is related to its concentration in brain. The possible mechanism is to enhance the inhibitory effect of γ-aminobutyric acid (GABA) by affecting the synthesis or metabolism of GABA.^[19]^ Initially, the patient was treated with sodium valproate, but symptoms were not controlled. This might be due to poor blood brain barrier penetration of sodium valproate, therefore limited its efficacy in epilepsy. Carbamazepine may limit the release of presynaptic and postsynaptic neuronal action potentials by increasing the efficacy of sodium channel inactivation, limiting postsynaptic neurons and blocking presynaptic sodium channels, blocking the release of excitatory neurotransmitters and reducing neuronal excitability.^[20]^ However, it is a CYP3A4 liver enzyme inducer, which can reduce the concentration of tacrolimus. Levetiracetam has a weak interference on cytochrome P450 enzyme, and hardly affects the blood concentration of tacrolimus. At last, this drug was used to control epilepsy, and follow-up for 4 months, the epilepsy never occurred.

## 4 Conclusion

In summary, we report a case of tacrolimus-induced epilepsy with PMN, which emphasizes that history of cerebral vascular injury, hypertension, hypoproteinemia, and interacting drugs might contribute to the development of epilepsy with tacrolimus administration in these patients.

## Acknowledgment

The authors thank Jie Zhang for English language editing. Jie Zhang is a PhD student at Aarhus University. She received her master of medicine (equivalent to MD degree) at Fudan University and master of public health degree at Brown University.

## Author contributions

**Data curation:** Rong Ren.

**Methodology:** Ying Mo.

**Project administration:** Fengmei Wang.

**Writing – original draft:** Yan Yang.

**Writing – review & editing:** Lei Zhang, Fengmei Wang.
